# Efficient termination of transcription by RNA polymerase I requires a conserved hairpin of the ribosomal RNA precursor

**DOI:** 10.1126/sciadv.adw2470

**Published:** 2025-08-20

**Authors:** Soren Nielsen, Nikolay Zenkin

**Affiliations:** Centre for Bacterial Cell Biology, Biosciences Institute, Faculty of Medical Sciences, Newcastle University, Baddiley-Clark Building, Richardson Road, Newcastle Upon Tyne NE2 4AX, UK.

## Abstract

RNA polymerase I (Pol I) synthesizes ribosomal RNA precursor (pre-rRNA), which comprises most of RNA in eukaryotic cells. Despite decades of investigation, there is still no consensus on what causes Pol I transcription termination. Here, we show that efficient termination by Pol I, paused by termination roadblock protein, is caused by RNA hairpin of the nascent pre-rRNA. Hairpin-dependent termination takes place at a physiological rate and does not require trans-acting factors. The function of the roadblock protein and the T-rich sequence is to synergistically cause deep backtracking of Pol I toward the termination RNA hairpin. Simultaneously, Pol I is catalytically inactivated, preventing rescue from backtracking through RNA cleavage and thus committing Pol I to termination. Termination RNA hairpins are present in most of Pol I terminators of eukaryotes, suggesting conservation of the RNA hairpin–dependent mechanism of Pol I transcription termination. We propose a simple model that unifies previous findings.

## Teaser

Ribosomal RNA that comprises most of RNA in the cell dictates the end of its own synthesis.

## INTRODUCTION

**Fig. 1.**
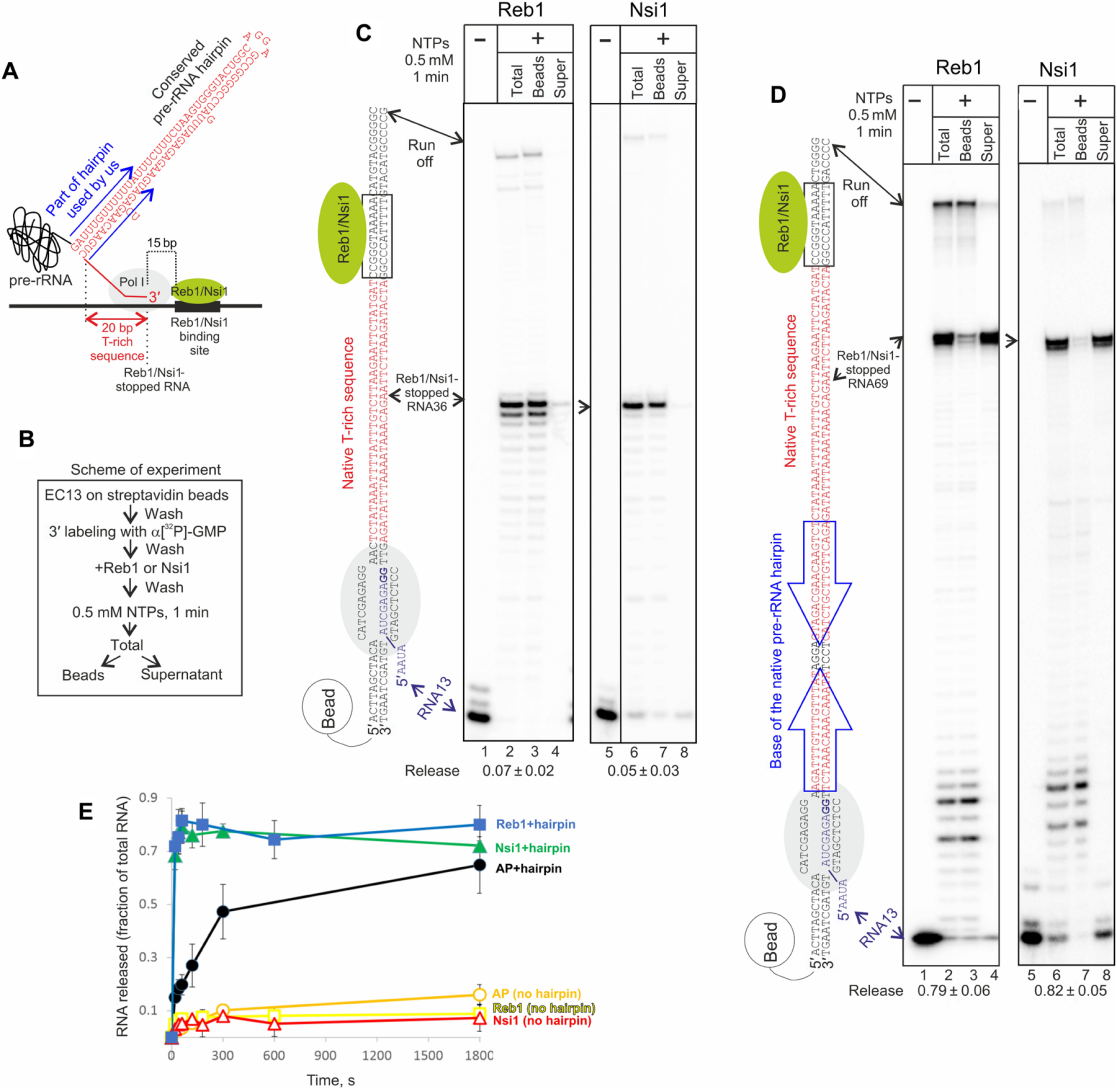
Conserved RNA hairpin of the pre-rRNA causes termination of stopped EC. (**A**) Scheme of the end of pre-rRNA gene, where Pol I terminates transcription after being stopped by Reb1 or Nsi1 roadblock. Blue arrows show the part of native hairpin used in the assembled ECs. Details of all ECs are shown in fig. S1B. (**B**) Scheme of the experiment for analysis of Pol I termination. Note that RNA13 in EC13 is radiolabeled at the 3′ end using the natural intrinsic cleavage activity of Pol I. (**C**) Reb1- or Nsi1-stopped Pol I does not terminate on the T-rich sequence. The scheme of the EC is shown next to the gel (EC^a^ in fig. S1B). Hereinafter, radioactive GMPs of the RNA (purple) are in bold, and quantitation of the RNA release is below the gels [super/(super + beads); means ± SD from at least three independent experiments]. (**D**) In the presence of the conserved pre-rRNA hairpin (termination hairpin), Reb1- or Nsi1-stopped EC efficiently and rapidly terminates transcription. The scheme of the EC is shown next to the gel (EC^c^ in fig. S1B). Blue arrows show the part of the termination hairpin coded in this EC [see (A)]. (**E**) RNA hairpin–dependent termination takes place at physiological rate. ECs were stopped by either Reb1, Nsi1, or AP at the position of the natural stop by Reb1/Nsi1, in the presence or absence of the termination hairpin (EC^a,b,c,d^ in fig. S1B). Note that the fastest time point possible in this experiment is 20 s. Data points are the mean of at least three independent experiments; error bars are ±SD. The raw data are presented in table S1.

Termination of transcription consists of pausing of the transcription elongation complex (EC), followed by its destruction. Termination of transcription by eukaryotic RNA polymerase I (Pol I) requires roadblock proteins conserved in all eukaryotes: Reb1 or Nsi1 in *Saccharomyces cerevisiae*, Reb1 in *Schizosaccharomyces pombe*, Rib2 in *Xenopus laevis*, or TTF1 in mammals (*1*–*4*). The roadblock protein binds to its binding site at the end of the pre-rRNA gene (Fig. 1A). Collision with the roadblock protein pauses transcription elongation within a T-rich (pyrimidine-rich in some eukaryotes; on the non–template strand) sequence 15 base pairs (bp) upstream of the roadblock binding site (Fig. 1A). However, the molecular pathway leading to destruction of the EC and release of the transcript, i.e., termination, remains debatable and contradictory. One model suggests that collision of Pol I with Nsi1 is enough to cause termination in *S. cerevisiae* (*5*, *6*). Another model in *S. cerevisiae* proposes that Reb1 and the T-rich sequence are required for transcript release (*7*, *8*). Similar contradictory models were proposed for *X. laevis* and mouse Pol I termination (*9*–*11*). Yet, another model in *S. cerevisiae* suggests that the T-rich sequence is enough for termination, independently of the roadblock (*12*). In *S. pombe*, however, the specific interaction with Reb1 was proposed to change properties of Pol I, which may be required for termination (*4*). A very different “torpedo” model in *S. cerevisiae* and *S. pombe* suggests involvement of in-trans-acting factors that cleave nascent pre-RNA upstream of the Nsi1- or Reb1-roadblocked EC and then exonucleolytically degrade the EC-bound part of the transcript until reaching the EC and destroying it (*13*–*15*). An alternative model in mammals suggests that termination requires a release factor PTRF in addition to the roadblock and the T-rich sequence (*16*).

## RESULTS

### Pol I termination is caused by pre-RNA hairpin

To investigate the details of the termination of transcription by Pol I, we used purified *S. cerevisiae* proteins (fig. S1A) and assembled ECs (fig. S1B; the EC identifiers are in the figure legends), which allowed investigation of the minimal requirements for termination. EC13, containing 13-nucleotide-long transcript (RNA13), was immobilized on streptavidin beads via biotin on the non–template strand, allowing exchange of the reaction components and analysis of RNA release as a result of termination (by separation of beads and supernatant fractions) (Fig. 1B). RNA13 was radiolabeled at the 3′ end using intrinsic Rpa12-mediated RNA cleavage of Pol I, which resulted in removal of 3′-end guanosine monophosphates (GMPs) and their replacement with α[^32^P]-GMPs through natural incorporation (lanes 1 and 5 of Fig. 1, C and D; bold Gs in schemes next to the gels). This ensured that only ECs containing active Rpa12, which was proposed to be important for termination (*4*, *17*), were analyzed. DNA downstream of the EC13 contained the native T-rich sequence and Reb1/Nsi1 binding site (compare schemes in Fig. 1, A and C). Reb1 or Nsi1 were added to the EC13, and an excess of non–bound protein was washed away. Termination was analyzed after 1 min of transcription by separation of beads and supernatant fractions (reaction scheme in Fig. 1B).

As expected, both Reb1 and Nsi1 efficiently stopped transcription 15 bp upstream of the Reb1/Nsi1 binding site, at the position corresponding to the 3′ end of native unprocessed pre-rRNA (*3*) (Fig. 1C, lanes 2 and 6). However, this did not result in termination of transcription, i.e., release of the RNA (Fig. 1C, lanes 3 and 4 and 7 and 8).

The T-rich sequence is naturally preceded by a sequence coding for a 36-bp-long (in *S. cerevisiae*) RNA hairpin (Fig. 1A). The experimental system did not allow introduction of the sequence coding for the whole 36-bp-long RNA hairpin due to interference of long secondary structures of the template and the non–template DNA strands with the EC assembly. Therefore, we introduced sequence coding for the 16-bp-long base of this hairpin in its native position (blue arrows in schemes in Fig. 1, A and D). As can be seen from Fig. 1D (lanes 3 and 4 and 7 and 8), transcription on this template resulted in efficient and rapid release of either Reb1- or Nsi1-stopped RNA, i.e., termination of transcription. The presence of a sequence of the similar length but not forming stable secondary structure upstream of the T-rich sequence did not cause termination (fig. S2A). Note that, in the absence of a roadblock protein, Pol I does not pause but transcribes to the end of the template (fig. S2B; “run off” product), and this product is not released (because the EC remains bound to the end of DNA; Fig. 1, C and D).

We analyzed kinetics of termination by measuring RNA release into the supernatant. In the absence of the termination RNA hairpin, ECs roadblocked by either Reb1 or Nsi1 remained intact even after 30-min incubation (Fig. 1E, yellow and red plots, respectively). In contrast, in the presence of the termination RNA hairpin, almost all RNA was released within 20 s (shortest time point experimental system allows), regardless of whether they were stopped by Reb1 or Nsi1 (Fig. 1E, blue and green plots, respectively). As Reb1 and Nsi1 behaved in the same way during termination, the following experiments were performed with Reb1.

The above results apparently contradict previous findings that roadblock alone (*5*, *6*, *9*) or T-rich sequence alone (*12*) or roadblock together with T-rich sequence (*6*–*8*, *10*, *11*) are sufficient for termination. We noted, however, that the templates used in those studies (*5*–*12*) contained orthologous sequences upstream of the T-rich sequence and/or the roadblock, which formed stable RNA secondary structures (mFold prediction; for example, fig. S2C). We designed ECs so that they would replicate constructs from (*5*) including sequences coding for most probable predicted RNA secondary structures upstream of the T-rich sequence (fig. S2C). Introduction of these sequences caused efficient termination (fig. S2C). Consistently, we found that the sequence of the termination RNA hairpin was not important because a random stable 9-bp-long RNA hairpin in place of the native one also caused efficient termination (fig. S2D).

The in vitro study of mouse Pol I termination suggested that a release factor PTRF was strictly required for RNA release (*16*). However, the termination RNA hairpin (see mouse terminators in fig. S3) in this study was replaced by an orthologous sequence that did not form stable RNA secondary structures (mFold prediction), suggesting a reason why the native RNA hairpin–dependent termination was not observed.

### Pol I backtracks toward termination RNA hairpin

**Fig. 2.**
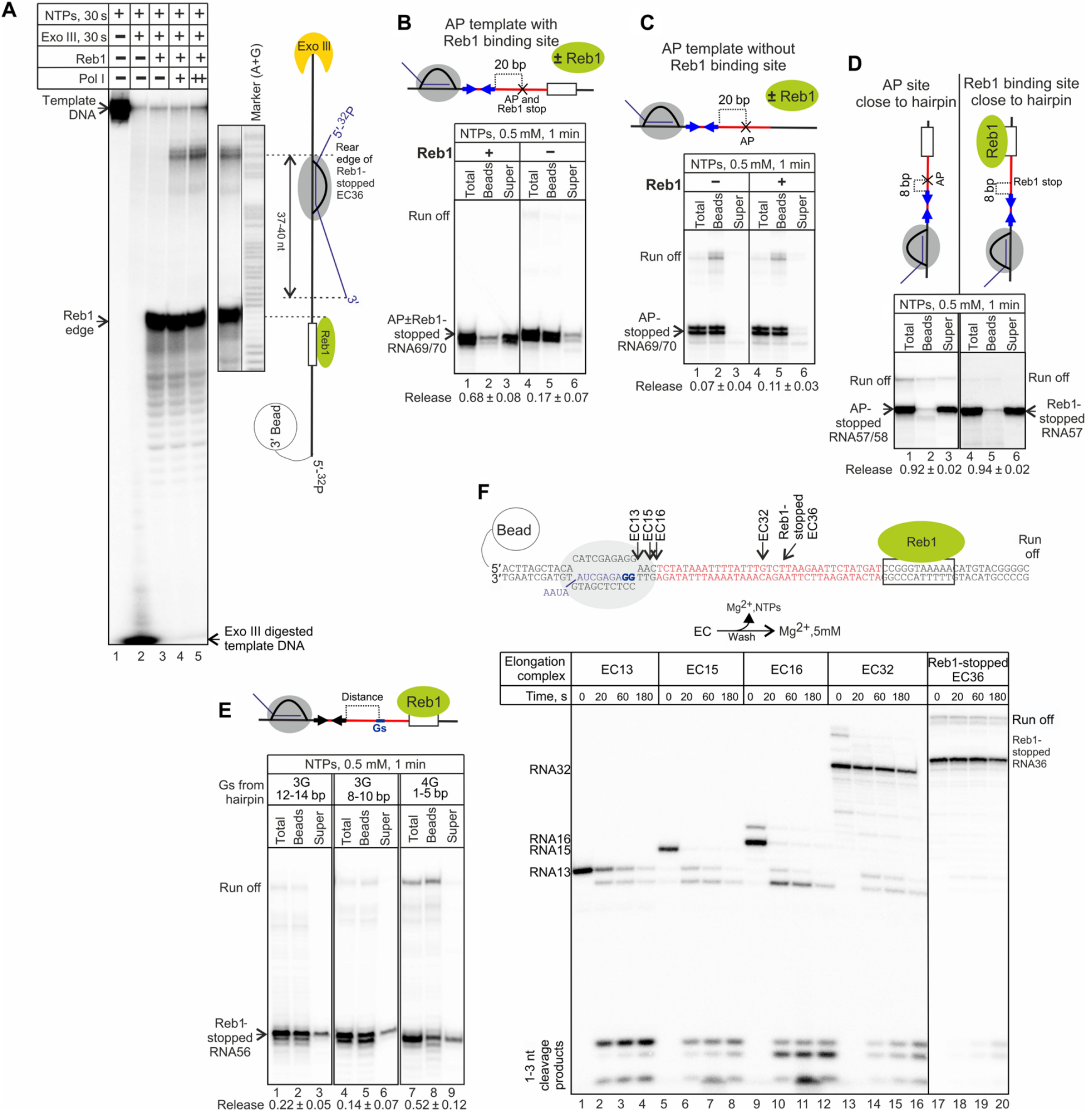
Reb1-stopped EC undergoes deep backtracking along the T-rich sequence with inactivation of RNA cleavage activity. (**A**) Exo III footprinting of the rear edge of the Reb1-stopped EC (EC^n^ in fig. S1B). The template DNA is radiolabeled at the 5′ end. The 3′ end of the non–template DNA contains phosphorothioate bonds to prevent Exo III digestion. The observed undersaturation of the Reb1-DNA complexes with the EC is expected from the way of EC assembly (see Materials and Methods). nt, nucleotides. (**B** and **C**) Stopping the EC by AP in the template DNA does not cause quick termination (see also Fig. 1E), which is restored by Reb1, but only when it is bound to its binding site (EC^d,e^ in fig. S1B). Note that Pol I misincorporates at the AP site, whereas addition of Reb1 abolishes this misincorporation, indicating correct binding on the AP template. (**D**) The roadblock and T-rich sequence are only required to efficiently bring the EC in the proximity of the termination RNA hairpin because rapid termination is achieved when the EC is stopped 8 bp downstream of the RNA hairpin by either AP or Reb1 (EC^f,g^ in fig. S1B). (**E**) Mutagenesis of the T-rich sequence inhibits termination, likely by affecting backtracking of the roadblocked EC toward the termination RNA hairpin (EC^l,m^ in fig. S1B) and strengthening of the RNA/DNA hybrid of terminating EC (EC^k^ in fig. S1B). The control with the native T-rich sequence is shown in fig. S2D. (**F**) Efficient cleavage activity of Pol I is inhibited during deep backtracking. ECs were one-step walked in subsets of NTPs to the positions indicated or stopped by Reb1 roadblock and washed, and cleavage was initiated by addition of Mg^2+^. Note that initial RNA13 was labeled at the 3′ end before walking (bold Gs in the scheme above; EC^a^ in fig. S1B).

**Fig. 3.**
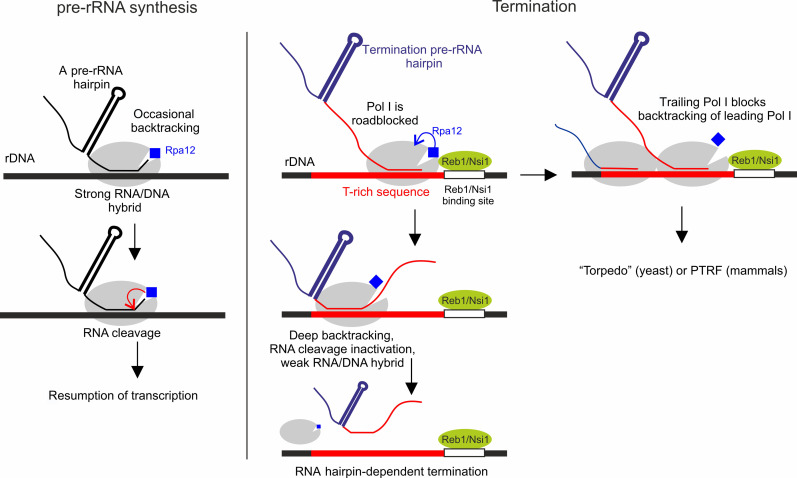
Model of termination by Pol I. Kinetics and functional discrimination between transcribing and terminating Pol I ECs. Note that the change in Rpa12 (blue) position is hypothetical and only shown to reflect inactivation of RNA cleavage activity. We suggest that RNA hairpin–dependent termination is the main termination pathway, which is further supported by fail-safe “torpedo” and release factor PTRF termination mechanisms when Pol I traffic jams may obstruct the RNA hairpin–dependent pathway (see Discussion).

The native termination RNA hairpin forms 20 nucleotides upstream of the 3′ end of the pre-rRNA. This distance is too large for the hairpin to affect the roadblocked EC directly (*18*) (for example, during bacterial termination, this distance is ~8 nucleotides). We hypothesized that the Reb1-stopped EC must undergo backtracking, which would bring it closer to the termination RNA hairpin. To determine the position of the EC after collision with Reb1, we used exonuclease III (Exo III) footprinting on the template strand that shows the position of the rear edge of the EC (scheme in Fig. 2A). The construct did not code for a hairpin (which, otherwise, would cause termination) and contained two phosphorothioate bonds at the 3′ end of the non–template strand to preclude its digestion by Exo III. In the absence of the EC, Exo III digestion was stopped by the bound Reb1 (Fig. 2A, lane 3). The Reb1-stopped EC blocked progression of Exo III upstream of the Reb1 (Fig. 3, lanes 4 and 5; undersaturation of the EC on Reb1-bound DNA was expected; see Materials and Methods). However, the rear edge of the roadblocked Pol I corresponded to a deeply backtracked EC, rather than an EC remaining near Reb1 (scheme in Fig. 2A).

### Native roadblock and T-rich sequence are required for efficient backtracking

To understand the role of Reb1 roadblock in termination, we stopped Pol I by introducing an abasic site (AP) into the DNA template at the position of the natural Reb1-dependent stop (Fig. 2B, lane 4; note that some misincorporation took place at the AP site resulting in two bands). As in the case of Reb1- or Nsi1-roadblocked ECs, we observed no termination in the absence of the RNA hairpin (Fig. 1E, orange plot). However, even in the presence of termination RNA hairpin, the AP-stalled EC was terminating much slower (>50 times) than Reb1- or Nsi1-roadblocked ECs (Fig. 1E, black plot; Fig. 2B, lanes 5 and 6). Binding of Reb1 to its binding site on the AP-template restored rapid RNA hairpin–dependent termination of the AP-stalled EC (Fig. 2B, lanes 2 and 3; note that the bound Reb1 blocks misincorporation at the AP site, confirming its correct binding). These results indicate that Reb1 is needed for efficient termination. Our results also explain the previous conclusion that, during termination, the T-rich sequence acts independently of the roadblock because termination of the Pol I EC stopped by the orthologous Lac repressor in that study was observed only after 30 min (*12*) (similar to the time required for termination on the AP template; see Fig. 1E). Addition of Reb1 in the absence of its binding site did not improve slow hairpin-dependent termination of the AP-stalled ECs (Fig. 2C), indicating that it must be bound to its binding site and cannot affect the EC “in-trans.”

The AP site disfavors backtracking of the AP-stalled EC (*19*), suggesting that Reb1 may be needed to initiate efficient backtracking. To test this hypothesis, we moved the AP site and Reb1/Nsi1 binding site so that the EC was stopped just 8 bp downstream of the termination RNA hairpin. Such arrangement would not require deep backtracking of the stopped EC to reach the termination RNA hairpin. As can be seen from Fig. 2D, when deep backtracking was not needed, both AP-stopped and Reb1-stopped ECs terminated equally efficiently. These results therefore suggest that Reb1 (and Nsi1) bound to the Reb1/Nsi1 binding site is required only for efficient backtracking of the EC toward the termination RNA hairpin. Although any other roadblock may lead to eventual backtracking and RNA hairpin–dependent termination, the times required for it are not physiological [for example, 30 min for AP in Fig. 1E and as much for the Lac repressor (*12*)]. This is also supported by an observation in vivo; orthologous bacterial protein LexA, when serving as the roadblock, did not support efficient termination by a hairpin coded by the neighboring upstream sequence at a distance requiring backtracking (*6*).

Our results suggest that the T-rich sequence may be important for backtracking of the EC toward the termination RNA hairpin and/or for the EC destruction. To disrupt backtracking, we introduced 3G-tracks (non–template strand) into the T-rich sequence at positions 12 to 14 and 8 to 14 bp downstream of the termination RNA hairpin. As can be seen from Fig. 2E (lanes 1 to 6), termination was diminished by either of the mutations (compare to the same construct with the native T-rich sequence in fig. S2D). To test the importance of a weak RNA/DNA hybrid, 4G-track was introduced at position 1 to 5 bp downstream of the termination RNA hairpin. This mutation also diminished termination, but to a lesser extent (Fig. 2E, lanes 7 to 9, and fig. S2D).

The findings apparently contradict previous studies that showed that the T-rich sequence is not required for termination (*5*, *9*). We, however, noticed that the templates used in these studies were constructed so that they contained orthologous RNA hairpins close to the roadblock and thus did not require backtracking. As we showed above, stopping the EC close to the RNA hairpin leads to efficient termination (Fig. 2D). Also, long incubation times used in these experiments (*5*, *9*) alleviated the requirement for a weak RNA/DNA hybrid that is needed only in physiological time frame (Fig. 2E, lanes 7 to 9).

### Deep backtracking inactivates Pol I RNA hydrolytic activity

Backtracking of an EC is commonly rescued by RNA cleavage in the active site of RNA polymerase, which restores transcript’s 3′ end in the active site. In Pol I, this cleavage reaction is catalyzed by the Rpa12 subunit and is highly efficient (as described above, we used this reaction to radiolabel RNA13 in the ECs, thus ensuring the presence of active Rpa12). Therefore, RNA in the ECs, backtracked after collision with Reb1, is expected to be cleaved. Earlier structural study has, however, suggested that long backtracking may cause rearrangement of the Pol I active center, potentially blocking cleavage activity (*20*). We analyzed RNA hydrolysis in ECs walked to different positions within the T-rich sequence (in subsets of NTPs) or roadblocked by Reb1. NTPs were removed before hydrolysis was initiated (scheme in Fig. 2F). As can be seen from Fig. 2F (lanes 1 to 12), Pol I quickly cleaved transcripts in EC13, EC15, and EC16. Cleavage of mono-, di-, or trinucleotides proceeded in a stepwise manner (until the radiolabeled α[^32^P]-GMPs at the positions 12 to 13 were reached and cleaved out, making the rest of the transcript invisible). However, cleavage activity in EC32 was strongly diminished (Fig. 2F, lanes 13 to 16). Cleavage in the roadblocked EC36 was even slower (Fig. 2F, lanes 17 to 20). The results indicate that deep backtracking switches off the hydrolytic activity of Pol I. We suggest that such catalytic transformation of deeply backtracked EC is critical to commit Pol I to reaching the termination RNA hairpin, instead of continuously being rescued from backtracking by RNA cleavage. Deep backtracking and switching off hydrolytic activity are likely to be parts of one and the same process (*20*) (see also Discussion).

Our results may explain a shorter RNA product in the roadblocked *X. laevis* Pol I ECs (*9*). In that study, the T-rich sequence was replaced by an orthologous sequence, which would not support deep backtracking and switching off the cleavage activity upon collision with roadblock (Rib2). Instead, this would lead to a gradual cleavage off the RNA 3′ end, similar to the results above (Fig. 2F, lanes 1 to 12) and the recent observation with stopped *S. cerevisiae* Pol I EC containing a long transcript of non–U-rich sequence (*21*).

## DISCUSSION

The principal finding of this work is that efficient termination of transcription by Pol I requires, along with the roadblock protein and T-rich sequence, a conserved RNA hairpin of the nascent pre-rRNA. RNA hairpin–dependent termination takes place at a physiologically relevant rate and does not require any additional trans-acting factors. Stable RNA hairpins are found upstream of the roadblocks and the pyrimidine-rich elements of Pol I terminators in *S. cerevisiae*, *S. pombe*, *X. laevis*, mouse, and humans (fig. S3), suggesting that the RNA hairpin–dependent termination of Pol I transcription is conserved among eukaryotes. Notably, most of the fail-safe terminators downstream the main ones also contain RNA hairpins upstream of the roadblock binding sites (fig. S3). These hairpins are not involved in maturation of pre-rRNA, suggesting their dedicated role in termination. The fail-safe terminator of *S. cerevisiae* does not contain a recognizable Nsi1/Reb1 binding site (*22*), although containing the T-rich sequence preceded by the stable RNA structure. This suggests that other roadblocks (such as, for example, nucleosomes) or yet unknown mechanisms may be involved in fail-safe termination of Pol I transcription in vivo. Below and in Fig. 3, we summarize our findings, explain how they solve discrepancies between the previous models, and propose a unified model that includes previous findings.

The requirement for the roadblock protein for Pol I termination is agreed in the field. However, some uncertainty remained, whether Reb1 or Nsi1 serve as a roadblock in *S. cerevisiae* (*5*, *7*). Our results reveal that both proteins can act in termination with the same efficiency in vitro. We, however, cannot exclude the possibility that selectivity for Nsi1 at termination sites (*6*) may imply a specific effect of Nsi1 for termination in vivo.

We found that the collision with Reb1 is followed by deep backtracking of the EC. At the same time, RNA cleavage activity of Pol I, which can rescue backtracked EC, is switched off, thus committing the EC to termination. The Rpa12 subunit that catalyzes RNA cleavage also physically blocks deep backtracking (*20*). We suggest that switching off the cleavage activity and deep backtracking are parts of one and the same event that involves displacement of Rpa12 from its normal position in the active center, as was suggested previously (*20*). Our results indicate that the native roadblock proteins may play an active role in it, either (i) by “pushing” Pol I into deep backtracking, in which case the 3′ end of RNA displaces Rpa12 and leads to the turning off of the cleavage activity, or (ii) by interacting with Rpa12 to change its orientation and open up the way for RNA’s 3′ end for deep backtracking and, at the same time, turning off the cleavage activity. A genetic link between Reb1 and Rpa12 and involvement of Rpa12 in termination were shown previously (*4*, *17*).

The importance of the T-rich element in termination has been shown before (*8*, *12*), although the mechanism was not clear [contradictory results (*5*, *9*) were explained above]. Our results suggest that the T-rich element acts synergistically with the roadblock protein to force irreversible backtracking of the EC, thus committing the EC to termination and bringing it closer to the termination RNA hairpin. Simultaneously, by stimulating irreversible backtracking, the T-rich sequence also reduces the chance of reading through the roadblock, explaining the earlier observations that mutagenesis of the T-rich sequence causes roadblock read-through (*8*). The second role of the T-rich sequence is to weaken the RNA/DNA hybrid of the terminating EC, required for quick termination.

Catalytically inactivated EC backtracks toward the termination RNA hairpin where it is disassembled (Fig. 3). The involvement of an RNA hairpin in termination by Pol I explains discrepancies in the previous observations that did not consider the importance of the upstream sequences and either replaced them with orthologous sequences that coded for the stable secondary RNA structure that caused termination (*5*–*12*), or, instead, coded for the unstructured transcript that prevented termination (*16*). Disassembly of the EC likely takes place via an allosteric action of RNA hairpin on Pol I, as in the case of bacterial RNA polymerase termination (*23*). A force from the RNA hairpin on the EC is unlikely to be involved because the backtracked EC can freely move along the template.

Because stopping EC close to the termination RNA hairpin leads to efficient termination without need for backtracking (Fig. 2D), we suggest that rapid irreversible backtracking, caused by the roadblock protein and T-rich sequence, is a checkpoint, at which the EC that must terminate is kinetically distinguished from the actively transcribing ECs (that can run away from RNA hairpins of the pre-rRNA) to prevent premature termination elsewhere within the pre-rRNA gene (Fig. 3). The requirement for a weak RNA/DNA hybrid for efficient termination further adds a functional checkpoint (Fig. 3), reducing the probability of premature RNA hairpin–dependent termination during pre-rRNA synthesis.

RNA hairpin–dependent termination by Pol I takes place at a physiological rate of 0.12 ± 0.04 s^−1^ (estimated from Fig. 1E), which is close to the highest in vivo rate of pre-rRNA synthesis (*24*). A trailing EC, however, may catch up with the leading roadblocked EC and thus inhibit its backtracking and termination (Fig. 3). Such queuing of the EC may also increase chances of reading through the roadblock via cooperation between polymerases (*25*). We suggest that “torpedo” (*13*–*15*) (in yeast) and PTRF-mediated (*16*) (in mammals) termination mechanisms evolved as fail-safe mechanisms to resolve such Pol I queuing (Fig. 3). As predicted by our model, deletion of Rnt1 (the endonuclease that initiates “torpedo” mechanism) leads to accumulation of Pol I at the terminator and increases read-through of the roadblock (*13*, *17*). Notably, neither Rnt1 nor PTRF are essential in vivo (*26*, *27*), as well as the requirement for PTRF for termination in vitro (*16*) was likely concluded because of the absence of the termination RNA hairpin (as described above).

## MATERIALS AND METHODS

### Proteins

#### Pol I

Pol I was purified from *S. cerevisiae* strain GPY2, which contains plasmid pAS22 with the hexahistidine tagged and HA (hemagglutinin)–tagged Pol I subunit Rpa43 (*28*). The strain was grown in multiple 5-liter baffled conical flasks at 30°C in 2 x YPD (yeast extract, peptone, and dextrose) medium to OD_600_ (optical density at 600 nm) = 4 to 5. Yeast paste (600 g) was mixed 1:1 with buffer [400 mM tris-HCl (pH 7.9); 10% glycerol, 1 M (NH_4_)_2_SO_4_, 10 mM dithiothreitol (DTT), 8 mM EDTA, 1 mM phenylmethylsulfonyl fluoride (PMSF), 1 mM 4-(2-aminoethyl)benzenesulfonyl fluoride hydrochloride (AEBSF), 2 mM benzamidine, 10 μM pepstatin, and aprotinin (4 μg/ml); and 14 μM E-64 and 10 μM leupeptin]. Pol I was purified using a procedure modified from (*29*–*31*). Homogenization was achieved using a bead beater (Biospec) at 0°C. The lysate was centrifuged at 27,000*g* for 40 min. A cloudy supernatant was withdrawn and centrifuged at 120,000*g* for 90 min. The clear interphase between lipids at the top and pellet at the bottom was removed and precipitated by increasing (NH_4_)_2_SO_4_ to 1.37 M at +4°C for 60 min. The pellet was discarded, and the supernatant was precipitated overnight by increasing (NH_4_)_2_SO_4_ to 2.75 M. The precipitate was resuspended in buffer A [40 mM Hepes (pH 7.8), 10% (w/v) glycerol, 4 mM DTT, 2 mM EDTA, 2 mM PMSF, and 2 mM benzamidine)]. The conductivity was adjusted by dilution, to equal that of buffer A with 100 mM KCl and applied to a 400-ml Bio-Rex 70 (Bio-Rad) packed column. The column was washed with buffer B [40 mM Hepes (pH 7.8) and 10% (w/v) glycerol] containing 100 mM KCl and eluted with buffer B containing 600 mM KCl. The eluate was applied to a 250-ml Ni-Sepharose (Cytiva) column. The protein was eluted with buffer B containing 200 mM KCl and 250 mM imidazole. The eluate was applied to a 170-ml Heparin-Sepharose (Cytiva) column. Pol I was eluted with linear gradient in buffer B (140 to 1000 mM KCl). Fractions with Pol I were pooled and diluted with buffer C (40 mM Hepes-KOH, 1 mM EDTA, 2 mM DTT, 20% glycerol, 1 mM PMSF, and 2 mM benzamidine) until the conductivity was the same as buffer C containing 120 mM KCl. This sample was applied to a Mono Q column 10/100 GL (Cytiva), equilibrated in buffer C with 120 mM KCl and Pol I eluted with a gradient from 120 mM to 1 M KCl in buffer C. Fractions with Pol I were pooled and concentrated using an Amicon 4-ml centrifugal device (MWCO, 30 kDa) to a protein concentration of ~1.2 mg/ml and stored at −80°C (see the SDS gel in fig. S1A). The identity of large subunits Rpa190 and Rpa135 and the presence of all Pol I subunits were confirmed by mass spectrometry.

#### Reb1

The *S. cerevisiae REB1* gene was cloned into pET-28a (Novagen) to express Reb1 with an N-terminal hexahistidine tag. Reb1 expression was induced in T7 Express cells (New England Biolabs) at OD_600_ = 0.6 with 1 mM isopropyl-β-d-thiogalactopyranoside (IPTG) for 18 hours at 21°C. Reb1 was purified using a procedure modified from (*7*, *32*). Cells were harvested by centrifugation and homogenized in a cell disruptor (Constant Systems, UK) in a buffer containing 50 mM tris-HCl (pH 8.0), 20% glycerol, 0.1% antifoam A, 1 x protease inhibitor cocktail (Merck), 1 M KCl, and 20 mM imidazole. The homogenate was centrifuged at 48,000*g*, and the supernatant was applied to a 25-ml Ni-Sepharose HP (Cytiva) column. The column was washed with buffer A [50 mM tris-HCl (pH 8.0), 10% glycerol, and 500 mM KCl] containing 20 mM imidazole and bound protein eluted with buffer A containing 270 mM imidazole. The eluate was dialyzed into buffer B [20 Hepes-KOH (pH 7.9), 2 mM EDTA, and 5 mM DTT] containing 200 mM KCl and applied to a 25-ml Heparin-Sepharose 6 FF (Cytiva) column. The column was washed with buffer B containing 400 mM KCl, and the protein was eluted with buffer B containing 800 mM KCl. Reb1 was dialyzed into buffer B containing 200 mM NaCl and 20% glycerol, concentrated to 2 mg/ml, and stored at −80°C.

#### Nsi1

The *S. cerevisiae NSI1* gene was cloned into pET-21a to express Nsi1 with an N-terminal hexahistidine tag. Expression of Nsi1 was induced in T7 Express cells (New England Biolabs) at OD_600_ = 0.6 with 1 mM IPTG for 24 hours at +18°C. Cells were harvested and homogenized, and Ni-Sepharose chromatography was performed as described for Reb1 above. Protein-containing fractions were pooled and dialyzed into buffer A [50 mM tris-HCl (pH 8.0) and10% glycerol] containing 450 mM NaCl, 2 mM EDTA, and 5 mM DTT. The dialysate was applied to a 25-ml Heparin-Sepharose 6 FF (Cytiva) column equilibrated in the same buffer. The bound protein was eluted with buffer A containing 2 M NaCl, 2 mM EDTA, and 5 mM DTT. Fractions containing Nsi1 were concentrated and loaded on a HiLoad 16/600 Superdex 200 pg column (Cytiva). Size exclusion chromatography was run in buffer B [40 mM tris-HCl (pH 7.4), 600 mM NaCl, 2 mM EDTA, and 5 mM DTT]. Nsi1 was dialyzed into buffer B containing 20% glycerol, concentrated to 1 mg/ml, and stored at −80°C.

### Transcription

Artificial ECs were used to study termination of transcription previously (*33*, *34*). For an average five-reaction experiment, ECs were assembled with DNA and RNA oligonucleotides (IDT) (see fig. S1B for sequences) in 10 μl of transcription buffer (TB) [20 mM tris-HCl (pH 7.9), 40 mM NaCl, and 0.1 mM EDTA]. RNA13 (1 pmol) was heated with 1 pmol of template DNA to 70°C and slowly cooled down to room temperature. Pol I (0.4 pmol) was added for 10 min at 20°C, followed by addition of 5 pmol of 5′ or 3′-biotinylated non–template DNA for 10 min at 30°C. Complexes were immobilized on streptavidin beads (5-μl slurry; Cytiva) and washed with 0.8 ml of TB+200 mM NaCl and then two times with 0.8 ml of TB, and the volume was adjusted to 24 μl. RNA13 was radiolabeled by addition of 4 μl of α-[^32^P]GTP (0.37 megabecquerel/μl; Hartmann Analytic) and MgCl_2_ (5 mM final) for 1 min at 30°C. This resulted in removal of one or two GMPs from the 3′ end of RNA13 by Pol I Rpa12-mediated hydrolytic activity and incorporation of α-[^32^P]GMPs in their place. The labeling was stopped by washing complexes with 800 μl of TB+5 mM EDTA and then washing twice with 800 μl of TB to a final volume of 55 μl. Reactions (10 μl) were distributed into individual tubes, and where indicated, 2 pmol of Reb1 or Nsi1 was added for 5 min at 30°C. Access to the roadblock protein was removed by washing with TB (except for the experiment in Fig. 2C), and reaction volumes were adjusted to 8 μl. All further reactions were performed at 30°C. Transcription was initiated by addition of 1 μl of NTPs and 1 μl of MgCl_2_ to final concentrations of 0.5 and 5 mM, respectively, and allowed to proceed for 1 min unless stated otherwise.

For analysis of termination, the reaction was either stopped with an equal volume (10 μl) of formamide containing stop buffer (“total”) or separated into “beads” and “supernatant” fractions. The bead fraction was washed with 200 μl of TB and adjusted with stop buffer to 20 μl. Seven microliters of the supernatant fraction received an equal volume of stop buffer.

For analysis of kinetics of RNA release (Fig. 1E), 3 μl of the supernatant was taken at times indicated in Fig. 1E and supplied with an equal volume of stoppage buffer.

For RNA hydrolysis reactions (Fig. 2F), assembled ECs were supplied with the sets of NTPs, allowing them to transcribe only to positions shown in Fig. 2F. Reactions were washed with 800 μl of TB+5 mM EDTA and then twice with 800 μl of TB to a final volume of 38 μl. Hydrolysis was initiated by addition of 2 μl of MgCl_2_ (5 mM final concentration). Samples (3.5 μl) were withdrawn at times indicated in the figure and stopped with an equal volume of stoppage buffer.

For Exo III footprinting, template DNA was ^32^P labeled at the 5′ end with T4 polynucleotide kinase (New England Biolabs) before EC assembly (*35*). ECs were assembled as above, except that, for one reaction, 25 pmol of the template, 25 pmol of RNA13, 4 or 8 pmol of Pol I, 50 pmol of nontemplate, and 100 pmol of Reb1 were used. Twenty units of Exo III (New England Biolabs) were added for 1 min to the reaction after 30 s of transcription in the presence of 0.5 mM NTPs and 5 mM MgCl_2_ for 1 min. Reactions were stopped with an equal volume of stop buffer.

Products of all above reactions were resolved in 10 to 23% denaturing polyacrylamide gels, visualized by phosphorimaging using Typhoon (Cytiva), and analyzed using ImageQuant software (Cytiva). All reactions were performed at least three times.

## Supplementary Materials

### This PDF file includes:

Figs. S1 to S3 Table S1 References
