# Supplementary material for: Efficient termination of transcription by RNA polymerase I requires a conserved hairpin of the ribosomal RNA precursor
Source: Sci Adv. 2025 Aug 20;11(34):eadw2470. doi: 10.1126/sciadv.adw2470 (PMC12366685; doi:10.1126/sciadv.adw2470)
Supplement: Supplementary file 1 — Figs. S1 to S3 Table S1 References [file sciadv.adw2470_sm.pdf]

Science Advances
AAAS

Supplementary Materials for

Efficient termination of transcription by RNA polymerase I requires a conserved hairpin of the ribosomal RNA precursor

Soren Nielsen and Nikolay Zenkin

Corresponding author: Nikolay Zenkin, n.zenkin@ncl.ac.uk

Sci. Adv. **11**, eadw2470 (2025)
DOI: 10.1126/sciadv.adw2470

This PDF file includes:

Figs. S1 to S3
Table S1
References

Figure S1 (2 pages). Proteins and assembled ECs used in the study. A. SDS gels of purified Pol I, Nsi1 and Reb1. Identity of large Pol I subunits Rpa190 and Rpa135 and the presence of all Pol I subunits were confirmed by mass-spectrometry. **B.** The framed scheme shows the region of Pol I termination with colors corresponding to colors of ECs below: native T-rich and termination RNA hairpin sequences are in red; colored arrows show the sequence coding for RNA hairpins (native hairpin - blue, artificial – black, from previous study – green, cyan – top of native hairpin).

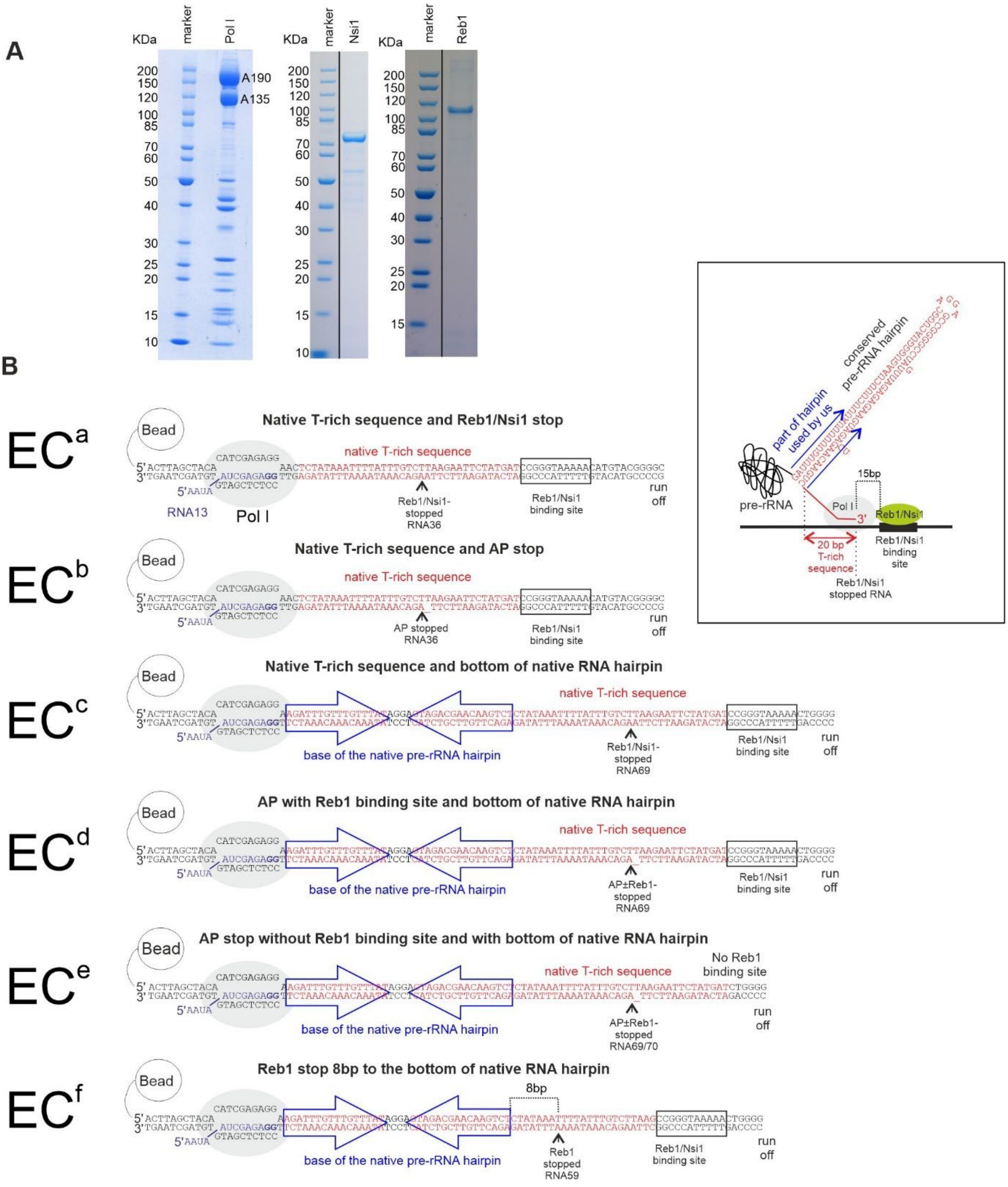

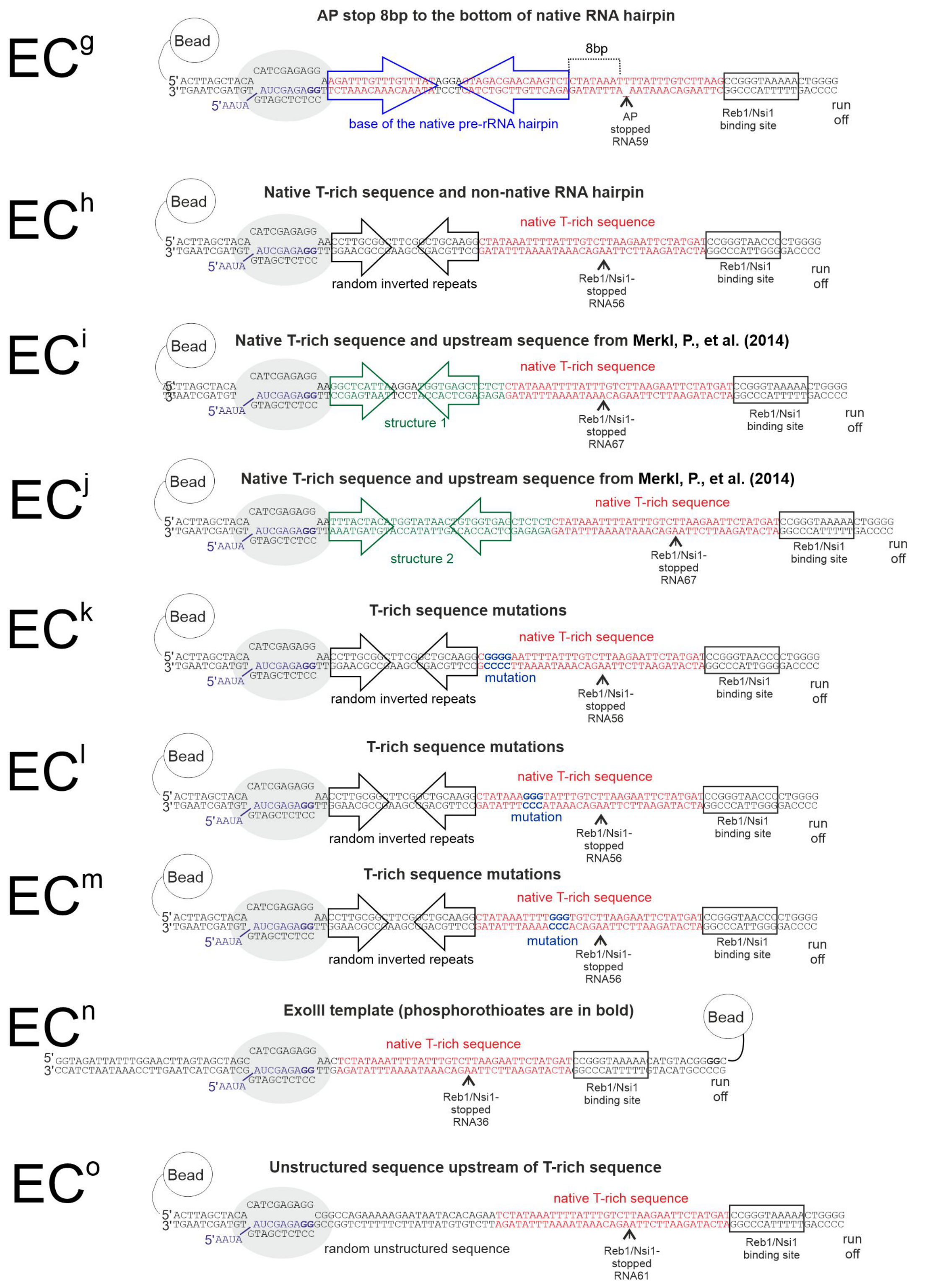

ECg
AP stop 8bp to the bottom of native RNA hairpin
Bead
8bp
base of the native pre-rRNA hairpin
AP stopped RNA59
Reb1/Nsi1 binding site
run off
ECh
Native T-rich sequence and non-native RNA hairpin
native T-rich sequence
random inverted repeats
Reb1/Nsi1-stopped RNA56
Reb1/Nsi1 binding site
run off
ECi
Native T-rich sequence and upstream sequence from Merkl, P., et al. (2014)
native T-rich sequence
structure 1
Reb1/Nsi1-stopped RNA67
Reb1/Nsi1 binding site
run off
ECj
Native T-rich sequence and upstream sequence from Merkl, P., et al. (2014)
native T-rich sequence
structure 2
Reb1/Nsi1-stopped RNA67
Reb1/Nsi1 binding site
run off
ECk
T-rich sequence mutations
native T-rich sequence
mutation
random inverted repeats
Reb1/Nsi1-stopped RNA56
Reb1/Nsi1 binding site
run off
ECl
T-rich sequence mutations
native T-rich sequence
mutation
random inverted repeats
Reb1/Nsi1-stopped RNA56
Reb1/Nsi1 binding site
run off
ECm
T-rich sequence mutations
native T-rich sequence
mutation
random inverted repeats
Reb1/Nsi1-stopped RNA56
Reb1/Nsi1 binding site
run off
ECn
ExoIII template (phosphorothioates are in bold)
native T-rich sequence
Reb1/Nsi1-stopped RNA36
Reb1/Nsi1 binding site
run off
ECo
Unstructured sequence upstream of T-rich sequence
native T-rich sequence
random unstructured sequence
Reb1/Nsi1-stopped RNA61
Reb1/Nsi1 binding site
run off

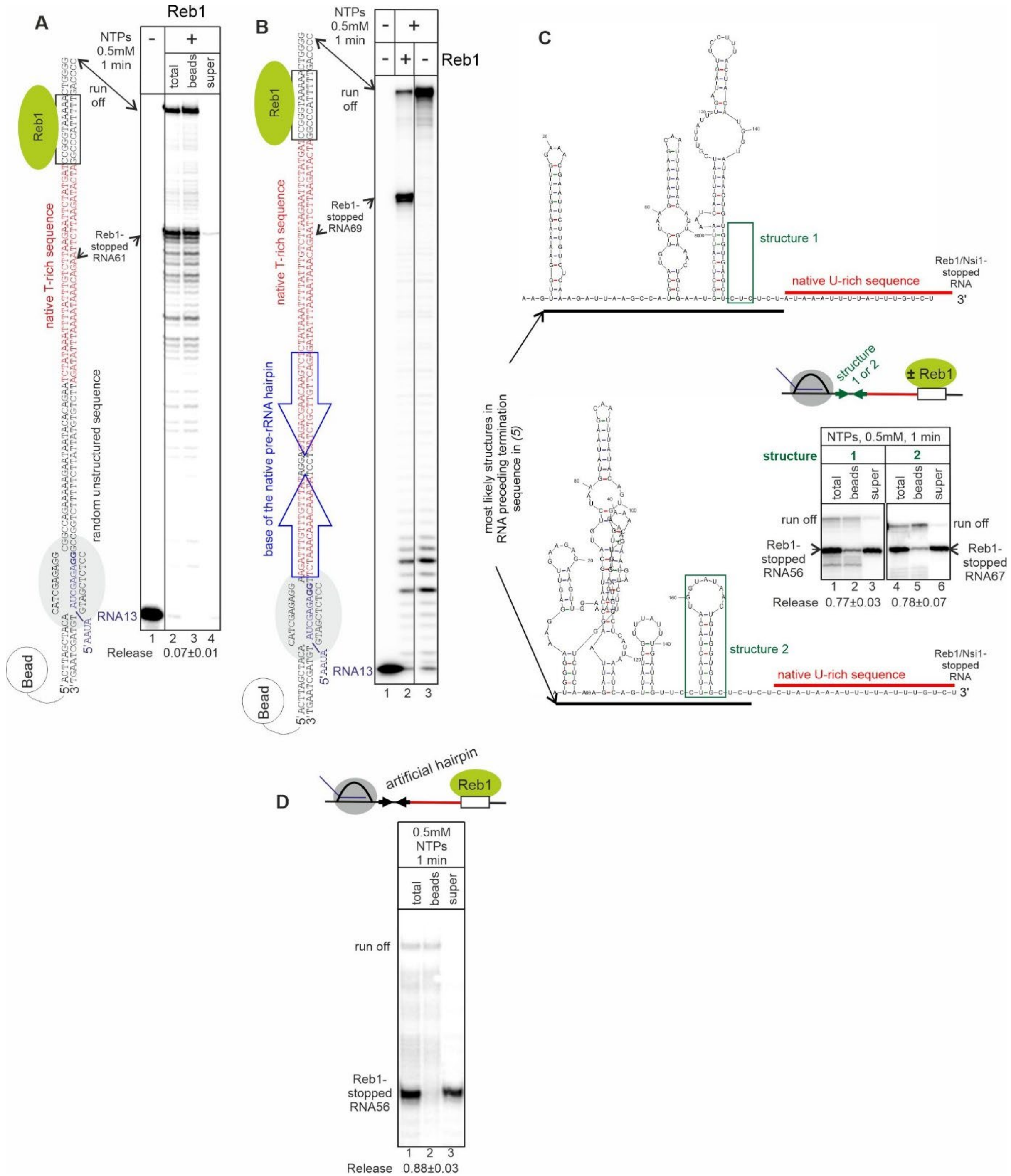

Figure S2. Characterization of termination by Pol I. A. Random region upstream of the T-rich sequence not coding for RNA secondary structure does not cause termination (EC^{o} in Fig. S1B). **B**. In the absence of roadblock proteins the EC transcribes without pausing to the end of the template (run off). **C**. Introduction of the most likely (according to mFold) secondary structures coded by orthologous sequences upstream of the T-rich sequence in the previous study (*5*) cause efficient termination (EC^{i,j} in Fig. S1B). **D**. Sequence of the termination RNA hairpin is not important, as artificial RNA hairpin at the position of the native pre-rRNA hairpin also causes efficient termination (EC^{h} in Fig. S1B).

Figure S3 (3 pages). Organization of terminators of pre-rRNA genes in eukaryotes. Binding sites of roadblock proteins are shown in red, and the predicted RNA hairpins are shown in yellow; alternative secondary structure marked in *Italic* or underlined. Arrows mark the experimentally determined termination sites (*16, 36-38*). Note that RNA hairpin is separated from the roadblock binding site by T-rich sequence. Sometimes “red” binding sites overlap with “yellow” RNA hairpin sequences of the next terminator.

Schizosaccharomyces pombe

```
gaagagatga agaagaagaa ggaggagagg aggaaggggg aagggggaag aagagggaga
cttctctact tcttcttctt cctcctctcc tccttccccc ttcccccttc ttctccctct

gaagagaaaa agtcgagcga gtcgattttt tttttggtct gaactttttc cctatatata
cttctctttt tcagctcgct cagctaaaaa aaaaaccaga cttgaaaaag ggatatatat

                                                        Reb1p
tataagagta tatagtatat ttataccttt gaaatatttt gaagagataa aaggtaaggg   T1
atattctcat atatcatata aatatggaaa ctttataaaa cttctctatt ttccattccc

taatgcactt ttgaagaaaa aaataaattc aaagtccaca cacgcagaca cttttttttg
attacgtgaa aacttttttt tttatttaag tttcaggtgt gtgcgtctgt gaaaaaaaac

tagtaggggg ggtattagta gtagtagtag tattagtatt aggaattggt gggaaaatac
atcatccccc ccataatcat catcatcatc ataatcataa tccttaacca cccttttatg

                                                         Reb1p
tcaataccca atacccaatt ttgtggaaaa gtaaaaactt tttgaaggga aaagaggtaa   T2
agttatgggt tatgggttaa aacacctttt catttttgaa aaacttccct tttctccatt

gggtaatgca caaatcctag taaaaaaaat
cccattacgt gtttaggatc atttttttta
```

Xenopus laevis

```
ctctcccgca ggaagggggc cgaaggcccc ttcggccccc gcaccccccc tccctcccgc
gagagggcgt ccttcccccg gcttccgggg aagccggggg cgtggggggg agggagggcg

              Rib2
cgggggaggc ctgacttgca ggcccgccgc gggacccctc ccggggagcg gggggggggg   T2
gccccctccg gactgaacgt ccgggcggcg ccctggggag ggcccctcgc cccccccccc
```

....

```
gctcgggcag agggagcagg ctcgtccccc tgccctgcag ccccaccggg agttccagga
cgagcccgtc tccctcgtcc gagcaggggg acgggacgtc ggggtggccc tcaaggtcct

gctcgggcag ggggagccgg ctcgtccccc ggcaccggag gtccccgggc cctttggcgc
cgagcccgtc cccctcggcc gagcaggggg ccgtggcctc caggggcccg ggaaaccgcg

                                   Rib2
ccgttttttc gcaaagtgcg gcgcccgcgg ggacttgctc ggccgggccg ggccccggcg   T3
ggcaaaaaag cgtttcacgc cgcgggcgcc cctgaacgag ccggcccggc ccggggccgc
```

Mouse (T7 does not contain stable RNA secondary structure upstream of roadblock, unless it is shared with T6. T3 and T4 may also share the same RNA secondary structure)

```
aacgccgcgt tcggcgccag gcctctggcg gccggggggg cgctctctcc gcccgagcat
ttgcggcgca agccgcggtc cggagaccgc cggccccccc gcgagagagg cgggctcgta

ccccactccc gcccctcctc ttcgcgcgcc gcggcggcga cgtgcgtacg aggggaggat
ggggtgaggg cggggaggag aagcgcgcgg cgccgccgct gcacgcatgc tcccctccta

gtcggggtgt ggaggcggag agggtccggc gcggcgcctc ttccattttt tcccccccaa
cagccccaca cctccgcctc tcccaggccg cgccgcggag aaggtaaaaa agggggggtt
                        TTF-I
cttcggaggt cgaccagtac tccgggcgac actttgtttt ttttttttcc cccgatgctg   T1
gaagcctcca gctggtcatg aggcccgctg tgaaacaaaa aaaaaaaagg gggctacgac
                TTF-I
gaggtcgacc agatgtccga aagtgtcccc cccccccccc ccccccgggg cggagcggcg   T2
ctccagctgg tctacaggct ttcacagggg gggggggggg ggggggcccc gcctcgccgc

gggccactct ggactctttt tttttttttt tttttttttt ttaaattcct ggaaccttta
cccggtgaga cctgagaaaa aaaaaaaaaa aaaaaaaaaa aatttaagga ccttggaaat
            TTF-I                                   TTF-I
ggtcgaccag ttgtccgtct tttactcctt catataggtc gaccagtact ccgggtggta   T3/T4
ccagctggtc aacaggcaga aaatgaggaa gtatatccag ctggtcatga ggcccaccat
                                         TTF-I
ctttgtcttt ttctgaaaat cccagaggtc gaccagatat ccgaaagtcc tctctttccc   T5
gaaacagaaa aagactttta gggtctccag ctggtctata ggctttcagg agagaaaggg

tttactcttc cccacagcga ttctcttttt tttttttttt tttggtgtgc ctctttttga
aaatgagaag gggtgtcgct aagagaaaaa aaaaaaaaaa aaaccacacg gagaaaaact
                                                            TTF-I
cttatataca tgtaaatagt gtgtacgttt atatacttat aggaggaggt cgaccagtac   T6
gaatatatgt acatttatca cacatgcaaa tatatgaata tcctcctcca gctggtcatg
                                                           TTF-I
tccgggcgac actttgtttt tttttttttt tccaccgatg atggaggtcg accagatgtc   T7
aggcccgctg tgaaacaaaa aaaaaaaaaa aggtggctac tacctccagc tggtctacag

cgaaagtgtc ccgtcccccc cctccccccc ccgcgacgcg gcgggctcac tctggactct
gctttcacag ggcagggggg ggaggggggg ggcgctgcgc cgcccgagtg agacctgaga
                                                      TTF-I
tttttttttt tttttttttt tttaaatttc tggaacctta aggtcgacca gttgtccgtc   T8
aaaaaaaaaa aaaaaaaaaa aaatttaaag accttggaat tccagctggt caacaggcag

tttcactcat tcatataggt cgaccggtgg tactttgtct ttttctcaaa atcgcagagg   T9
aaagtgagta agtatatcca gctggccacc atgaaacaga aaaagagttt tagcgtctcc

tcgaccagat gtcagaaagt .........
agctggtcta cagtctttca .........

aagccctctc tgtccctgtc accgggggcc ctgtacgtct gaggccgagg gaaagctatg
ttcgggagag acagggacag tggcccccgg gacatgcaga ctccggctcc ctttcgatac

ggcgcggttt tctttcattg acctgtcggt cttatcagtt ctccgggttg tcaggtcgac   T0
ccgcgccaaa agaaagtaac tggacagcca gaatagtcaa gaggcccaac agtccagctg
```

```
cagttgttcc tttgaggtcc ggttcttttc gttatggggt catttttggg ccacctcccc
gtcaacaagg aaactccagg ccaagaaaag caataccccca gtaaaaaccc ggtggagggg
```

Human

```
ggctcgtccg ctccgggccg ggacggggtc cggggagcgt ggtttgggag ccgcggaggc
ccgagcaggc gaggcccggc cctgccccag gcccctcgca ccaaaccctc ggcgcctccg

gccgcgccga gccgggcccc gtggcccgcc ggtccccgtc ccgggggttg gccgcgcggc
cggcgcggct cggcccgggg caccgggcgg ccaggggcag ggcccccaac cggcgcgccg

gcggtggggg gccacccggg gtcccggccc tcgcgcgtcc ttcctcctcg ctcctccgca  T1
cgccaccccc cggtgggccc cagggccggg agcgcgcagg aaggaggagc gaggaggcgt
          TTF-I
cgggtcgacc agcgaaccgc gggtggcggg cggcgggcgg cgagccccac gggcgtcccc
gcccagctgg tcgcttggcg cccaccgccc gccgcccgcc gctcggggtg cccgcagggg
                                                       TTF-I
gcacccggcc gacctccgct cgcgacctct cctcggtcgg gcctccgggg tcgaccgcct  T2
cgtgggccgg ctggaggcga gcgctggaga ggagccagcc cggaggcccc agctggcgga
                                                  TTF-I
gcgcccgcgg gcgtgagact cagcggcgtc tcgccgtgtc ccgggtcgac cgcggccttc  T3
cgcgggcgcc cgcactctga gtcgccgcag agcggcacag ggcccagctg gcgccggaag

tccaccgagc ggcggtgtag gagtgcccgt cgggacgaac cgcaaccgga gcgtccccgt
aggtggctcg ccgccacatc ctcacgggca gccctgcttg gcgttggcct cgcaggggca
                              TTF-I
ctcgggtcggc acctccgggg tcgaccagct gccgcccgcg agctccggac ttagccggcg  T4
gagccagccg tggaggcccc agctggtcga cggcgggcgc tcgaggcctg aatcggccgc
                           TTF-I
tctgcacgtg tcccgggtcg accagcaggc ggccgccgga cgcagcggcg cacgcacgcg  T5
agacgtgcac agggcccagc tggtcgtccg ccggcggcct gcgtcgccgc gtgcgtgcgc

.........

ggccagggcg gccgggaggg ctccccggcc cggcgctgtc cccgcgtgtg tccttgggtt  T0
ccggtcccgc cggccctccc gaggggccgg gccgcgacag gggcgcacac aggaacccaa

gaccagaggg accccgggcg ctccgtgtgt ggctgcgatg gtggcgtttt tggggacagg
ctggtctccc tggggcccgc gaggcacaca ccgacgctac caccgcaaaa acccctgtcc
```

Table S1. Raw data for the plots in Fig. 1E.

REB (NO HARPIN)						
Time, s						
0	0	0	0	0	0	0
20	0.0497356	0.0607	0.04691	0.024098	0.072158	0.039476
40	0.0574349		0.055967	0.035378	0.104882	0.065207
60	0.0567896	0.062142	0.055607	0.051143	0.113556	0.079353
180	0.059678	0.062343	0.057797	0.046311	0.121592	0.108616
600	0.0636992	0.063794	0.057814	0.053009	0.131627	0.119707
1800	0.0713174	0.064624	0.06495	0.054641	0.138101	0.140405

REB+HAIRPIN				
Time, s				
0	0	0	0	0
20	0.614578	0.7442176	0.433963	0.69434
40	0.684737	0.8862037	0.468168	0.749069
60	0.70546	0.8453723	0.512885	0.820616
120	0.693188	0.7975565	0.495665	0.793064
300	0.74313	0.7759065	0.505548	0.808877
1800	0.667945	0.6252227	0.412135	0.659416

NSI (NO HARPIN)				
Time, s				
0	0	0	0	0
20	0.0759982	0.0091	0.00698	0.033536
40	0.0996451	0.013356	0.010427	0.07085
60	0.0974975	0.013933	0.011858	0.085545
120	0.0692402			
180		0.01757	0.014106	0.111195
300	0.0804949			
600		0.018843	0.016141	0.11981
1800	0.1054489	0.025127	0.023559	0.138893

NSI+HAIRPIN			
Time, s			
0	0	0	0
20	0.743348	0.6526598	0.75974
40	0.705118	0.7124796	0.840745
60	0.766967	0.8025098	0.876779
180	0.689265	0.8816622	0.829993
600	0.643627	0.807114	0.780779
1800	0.637277	0.7154598	0.81481

AP (NO HAIRPIN)			
Time, s			
0	0	0	0
20	0.0184868	0.028787	0.020925
40		0.028567	0.04072
60	0.0258902	0.03683	0.040653
120	0.0389439	0.05268	0.065932
300	0.0737638	0.103052	0.128589
1800	0.1103811	0.204911	0.165596

AP+HAIPRIN				
Time, s				
0	0	0	0	0
20	0.147569	0.1509893		0.151289
40	0.232484	0.176339	0.187824	0.135125
60	0.234335	0.2130416	0.211302	0.133813
120	0.380605	0.2396638		0.190626
300	0.615321	0.4266161		0.377539
1800	0.808637	0.6292546	0.646958	0.509815

REFERENCES AND NOTES

1. I. Bartsch, C. Schoneberg, I. Grummt, Purification and characterization of TTFI, a factor that mediates termination of mouse ribosomal DNA transcription. *Mol. Cell Biol.* **8**, 3891–3897 (1988).

2. B. McStay, R. H. Reeder, A DNA-binding protein is required for termination of transcription by RNA polymerase I in *Xenopus laevis*. *Mol. Cell Biol.* **10**, 2793–2800 (1990).

3. W. H. Lang, R. H. Reeder, The REB1 site is an essential component of a terminator for RNA polymerase I in *Saccharomyces cerevisiae*. *Mol. Cell Biol.* **13**, 649–658 (1993).

4. R. Jaiswal, M. Choudhury, S. Zaman, S. Singh, V. Santosh, D. Bastia, C. R. Escalante, Functional architecture of the Reb1-Ter complex of *Schizosaccharomyces pombe*. *Proc. Natl. Acad. Sci. U.S.A.* **113**, E2267–2276 (2016).

5. P. Merkl, J. Perez-Fernandez, M. Pilsl, A. Reiter, L. Williams, J. Gerber, M. Bohm, R. Deutzmann, J. Griesenbeck, P. Milkereit, H. Tschochner, Binding of the termination factor Nsi1 to its cognate DNA site is sufficient to terminate RNA polymerase I transcription in vitro and to induce termination in vivo. *Mol. Cell Biol.* **34**, 3817–3827 (2014).

6. A. Reiter, S. Hamperl, H. Seitz, P. Merkl, J. Perez-Fernandez, L. Williams, J. Gerber, A. Nemeth, I. Leger, O. Gadal, P. Milkereit, J. Griesenbeck, H. Tschochner, The Reb1-homologue Ydr026c/Nsi1 is required for efficient RNA polymerase I termination in yeast. *EMBO J.* **31**, 3480–3493 (2012).

7. W. H. Lang, B. E. Morrow, Q. Ju, J. R. Warner, R. H. Reeder, A model for transcription termination by RNA polymerase I. *Cell* **79**, 527–534 (1994).

8. W. H. Lang, R. H. Reeder, Transcription termination of RNA polymerase I due to a T-rich element interacting with Reb1p. *Proc. Natl. Acad. Sci. U.S.A.* **92**, 9781–9785 (1995).

9. P. Labhart, The *Xenopus* 9 bp ribosomal terminator (T3 box) is a pause signal for the RNA polymerase I elongation complex. *Nucleic Acids Res.* **23**, 2252–2258 (1995).

10. P. Labhart, Negative and positive effects of CpG-methylation on *Xenopus* ribosomal gene transcription in vitro. *FEBS Lett.* **356**, 302–306 (1994).

11. A. Kuhn, A. Normann, I. Bartsch, I. Grummt, The mouse ribosomal gene terminator consists of 3 functionally separable sequence elements. *EMBO J.* **7**, 1497–1502 (1988).

12. S. W. Jeong, W. H. Lang, R. H. Reeder, The release element of the yeast polymerase I transcription terminator can function independently of Reb1p. *Mol. Cell Biol.* **15**, 5929–5936 (1995).

13. A. El Hage, M. Koper, J. Kufel, D. Tollervey, Efficient termination of transcription by RNA polymerase I requires the 5′ exonuclease Rat1 in yeast. *Genes Dev.* **22**, 1069–1081 (2008).

14. P. Braglia, J. Kawauchi, N. J. Proudfoot, Co-transcriptional RNA cleavage provides a failsafe termination mechanism for yeast RNA polymerase I. *Nucleic Acids Res.* **39**, 1439–1448 (2011).

15. J. Kawauchi, H. Mischo, P. Braglia, A. Rondon, N. J. Proudfoot, Budding yeast RNA polymerases I and II employ parallel mechanisms of transcriptional termination. *Genes Dev.* **22**, 1082–1092 (2008).

16. P. Jansa, S. W. Mason, U. Hoffmann-Rohrer, I. Grummt, Cloning and functional characterization of PTRF, a novel protein which induces dissociation of paused ternary transcription complexes. *EMBO J.* **17**, 2855–2864 (1998).

17. E. M. Prescott, Y. N. Osheim, H. S. Jones, C. M. Alen, J. G. Roan, R. H. Reeder, A. L. Beyer, N. J. Proudfoot, Transcriptional termination by RNA polymerase I requires the small subunit Rpa12p. *Proc. Natl. Acad. Sci. U.S.A.* **101**, 6068–6073 (2004).

18. L. Tafur, Y. Sadian, N. A. Hoffmann, A. J. Jakobi, R. Wetzel, W. J. H. Hagen, C. Sachse, C. W. Muller, Molecular structures of transcribing RNA polymerase I. *Mol. Cell* **64**, 1135–1143 (2016).

19. J. Woodgate, H. Mosaei, P. Brazda, F. Stevenson-Jones, N. Zenkin, Translation selectively destroys non-functional transcription complexes. *Nature* **626**, 891–896 (2024).

20. C. Engel, S. Sainsbury, A. C. Cheung, D. Kostrewa, P. Cramer, RNA polymerase I structure and transcription regulation. *Nature* **502**, 650–655 (2013).

21. T. W. Turowski, E. Petfalski, B. D. Goddard, S. L. French, A. Helwak, D. Tollervey, Nascent transcript folding plays a major role in determining RNA polymerase elongation rates. *Mol. Cell* **79**, 488–503 e411 (2020).

22. R. H. Reeder, P. Guevara, J. G. Roan, *Saccharomyces cerevisiae* RNA polymerase I terminates transcription at the Reb1 terminator in vivo. *Mol. Cell Biol.* **19**, 7369–7376 (1999).

23. V. Epshtein, C. J. Cardinale, A. E. Ruckenstein, S. Borukhov, E. Nudler, An allosteric path to transcription termination. *Mol. Cell* **28**, 991–1001 (2007).

24. J. R. Warner, The economics of ribosome biosynthesis in yeast. *Trends Biochem. Sci.* **24**, 437–440 (1999).

25. V. Epshtein, E. Nudler, Cooperation between RNA polymerase molecules in transcription elongation. *Science* **300**, 801–805 (2003).

26. X. Li, Z. Zhong, R. Zhang, J. Zhang, Y. Zhang, S. Zeng, Q. Du, H. Wang, S. Zhang, L. Lu, M. Li, K. Long, Decoding the transcriptome of muscular dystrophy due to Ptrf deficiency using single-nucleus RNA sequencing. *FASEB J.* **37**, e22993 (2023).

27. M. Catala, B. Lamontagne, S. Larose, G. Ghazal, S. A. Elela, Cell cycle-dependent nuclear localization of yeast RNase III is required for efficient cell division. *Mol. Biol. Cell* **15**, 3015–3030 (2004).

28. S. Fath, P. Milkereit, A. V. Podtelejnikov, N. Bischler, P. Schultz, M. Bier, M. Mann, H. Tschochner, Association of yeast RNA polymerase I with a nucleolar substructure active in rRNA synthesis and processing. *J. Cell Biol.* **149**, 575–590 (2000).

29. P. Milkereit, P. Schultz, H. Tschochner, Resolution of RNA polymerase I into dimers and monomers and their function in transcription. *Biol. Chem.* **378**, 1433–1443 (1997).

30. F. D. Appling, D. A. Schneider, Purification of active RNA polymerase I from yeast. *Methods Mol. Biol.* **1276**, 281–289 (2015).

31. P. Tongaonkar, J. A. Dodd, M. Nomura, Purification and assay of upstream activation factor, core factor, Rrn3p, and yeast RNA polymerase I. *Methods Enzymol.* **370**, 109–120 (2003).

32. B. E. Morrow, Q. Ju, J. R. Warner, Purification and characterization of the yeast rDNA binding protein REB1. *J. Biol. Chem.* **265**, 20778–20783 (1990).

33. S. Nielsen, Y. Yuzenkova, N. Zenkin, Mechanism of eukaryotic RNA polymerase III transcription termination. *Science* **340**, 1577–1580 (2013).

34. Y. Yuzenkova, N. Zenkin, K. Severinov, Mapping of RNA polymerase residues that interact with bacteriophage Xp10 transcription antitermination factor p7. *J. Mol. Biol.* **375**, 29–35 (2008).

35. D. Temiakov, N. Zenkin, M. N. Vassylyeva, A. Perederina, T. H. Tahirov, E. Kashkina, M. Savkina, S. Zorov, V. Nikiforov, N. Igarashi, N. Matsugaki, S. Wakatsuki, K. Severinov, D. G. Vassylyev, Structural basis of transcription inhibition by antibiotic streptolydigin. *Mol. Cell* **19**, 655–666 (2005).

36. Y. F. Melekhovets, P. S. Shwed, R. N. Nazar, In vivo analyses of RNA polymerase I termination in *Schizosaccharomyces pombe*. *Nucleic Acids Res.* **25**, 5103–5109 (1997).

37. P. Labhart, Heteroduplex analysis of the Xenopus Rna-polymerase-I terminator. *Biochem. Biophys. Res. Commun.* **212**, 1082–1087 (1995).

38. R. Evers, I. Grummt, Molecular coevolution of mammalian ribosomal gene terminator sequences and the transcription termination factor Ttf-I. *Proc. Natl. Acad. Sci. U.S.A.* **92**, 5827–5831 (1995).
